# Simultaneous Identification and Quantification of 20 β-Receptor Agonists in Feed Using Gas Chromatography-Tandem Mass Spectrometry

**DOI:** 10.1371/journal.pone.0076400

**Published:** 2013-10-03

**Authors:** Jie Cheng, Shi Wang, Xiao-Ou Su

**Affiliations:** Institute of Quality Standards and Testing Technologies for Agro-products, Chinese Academy of Agricultural Sciences, Beijing, China; Wageningen UR Livestock Research, Netherlands

## Abstract

“Lean meat powder” is a class of toxic chemicals that have structures similar to that of β-adrenergic receptor agonists. At least 16 chemicals from this class have been specifically banned by the 176^th^ bulletin of the Chinese Department of Agriculture on breeding animals, and methods for monitoring the illicit use of β-agonists in animal feed are required. Herein, a method to quantify 20 β-agonists in feed, via analyte derivatization followed by gas chromatography-tandem mass spectrometry, has been developed. The optimized method has a good linear correlation (calibration coefficient > 0.99) between the quantitative ion peak area and the concentration of β-agonists over a large working range (0.05–1 mg/kg). The limit of detection (LOD) was 0.01 mg/kg, the recoveries for three β-agonists spikes (0.05, 0.1, and 1 µg/g) in feed ranged from 75.6 to 102.4%, repeatability ranged from 1.2 to 9.4% for all of the compounds, and intermediate precisions were lower than 13.8%. This precise, accurate method was applied to quantify 20 β-agonists in actual feed samples and represents an excellent complement to existing quantification methods.

## Introduction

“Lean meat powder” is a class of compounds with structures similar to β-adrenergic receptor agonists (β-agonists). β-agonists are commonly used to treat asthma, bronchiectasis, and other respiratory diseases [1]. In animals, these compounds promote overall growth and enhance lean meat production, hence they are termed lean meat powder. Lean meat powder initially referred to clenbuterol hydrochloride, a β-agonist that significantly improved feed conversion rates and enhanced the percentage of lean meat in animals. When clenbuterol was banned, other β-agonists were used illicitly to circumvent regulation.

β-agonists were banned for use as growth promoters in livestock in the European Union in 1996 [2], and in 2002, the 176^th^ bulletin of the Chinese Department of Agriculture specifically prohibited the use of 40 drugs in feed and animal drinking water, including 16 β-agonists. To determine the concentrations of β-agonists in feed, the Chinese government relies mainly on the methods described in Bulletins 1063-6-2008 and 1063-7-2008 [3,4]. Even with tight regulation, the illegal use of β-agonists in animal feed continues because of financial incentives; see the Shuanghui event in Henan in March 2011 [5], the lijin sheep lean meat powder events in Shandong in 2011 [6], and the positive identification of β-agonists in imported beef in Taiwan in 2012 [7] for more details. For this reason, it is important to monitor the illicit use of β-agonists in animal feed.

Currently, the detection of β-agonists is based primarily on liquid chromatography-tandem mass spectrometry (LC-MS/MS) [8–10] and gas chromatography-mass spectrometry (GC-MS) [11–14] methods, as well as colloidal gold immunochromatography followed by rapid screening with enzymatic immune technology [15,16]. There are notable drawbacks to these methods: rapid screening methods are limited by high rates of “false positives”; instrumentation for LC-MS/MS, which has high sensitivity, is relatively expensive; current GC-MS methods, coupled with derivatizating agents such as BSTFA, MBA, and MBTFA, can only quantitate fewer than 9 individual β-agonists in feed [4,11–14], and no method to date has been reported for the simultaneous identification of 20 β-agonists. Additionally, the sensitivity of gas chromatography-mass spectrometry is relatively low, for example, the LOD of ractopamine in feed is 0.1 mg/kg [4].

In this study, gas chromatography-tandem mass spectrometry (GC-MS/MS) was performed on derivatized samples to simultaneous identify and quantify 20 β-agonists in feed; the samples were derivatized with (1) 1:1 (v:v) N-methyl-N-trimethylsilyltrifluoroacetamide (MSTFA)-ethyl acetate or (2) 1:1 (v:v) N,O-bis(trimethylsilyl) trifluoroacetamide containing 1% trimethylchlorosilane (BSTFA+TMCS)-toluene. This method offers comparable sensitivity and ruggedness as the prevailing LC-MS/MS method, detailed in Bulletin-1063-6-2008 [3], and can potentially provide technical support for regulating and monitoring β-agonists in animal feed.

## Materials and Methods

### Chemicals and materials

Standards of the 20 β-agonists were obtained from Sigma-Aldrich, USA. Sodium acetate buffer solution (pH = 4.8) was prepared in house. Oasis® MCX (60 mg/3mL, mixed-mode cation-exchange reversed-phase) and Oasis® HLB (60 mg/3mL, hydrophilic-lipophilic-balanced, water-wettable reversed-phase) solid phase extraction (SPE) columns were purchased from Waters, USA. A molecular imprinted (MIP) SPE column (β-agonists, 25 mg/10 mL) was purchased from Lund, Sweden. Chromatographically pure toluene and methanol were purchased from Merck, USA. All other analytical-grade reagents were obtained from the J&K company, Beijing, China. 99:1 (v:v) BSTFA+TMCS, MSTFA and methyl boronic acid (MBA; 10.2 mg/mL dissolved in ethyl acetate) were purchased from Fluka, USA. Blank samples were provided by the Chinese National Feed Control Center, and testing by LC-MS/MS proved that the 20 β-receptors were absent.

### Preparation of standard solutions

To prepare 200 mg/L stock solutions of the 20 β-agonists (purity > 99.5%), 20 mg of each β-agonist was accurately weighted out and dissolved in 100 mL of methanol. The stock solutions, stored in the dark at -20 °C for 3 months prior to analysis, were subsequently diluted with methanol.

### Sample extraction

Five grams of the feed sample was mixed with 50 mL sodium acetate buffer, and shaken by a mechanical shaker for 20 min at 200 rpm. The samples were allowed to stand for 3 min, the mixture was filtered rapidly through qualitative filter paper (φ = 12.5cm), and the filtrate was collected for further purification.

### Sample purification

The MCX SPE column was first activated with 3 mL of methanol and 3 mL of de-ionized water. Then, 2 mL of the extract filtrate was loaded onto the SPE column, and washed with 3 mL of 1 M acetic acid and 3 mL of methanol. The sample was eluted with 3 mL of 5% ammonium in methanol and dried by blowing N_2_ gas over the solution at 40 °C. The dried sample was then covered, placed in an oven at 70 °C for 5 min, and subsequently set in a desiccator to cool. The HLB and MIP SPE columns were activated using the same procedure.

### Sample derivatization

#### Derivatization with BSTFA and MSTFA

In a dry vial, 100 µL of toluene and 100 µL of 99:1 (v:v) BSTFA+TMCS or 100 µL ethyl acetate and 100 µL MSTFA was mixed. The vial was sealed and placed in an oven at 70 °C for 60 min. The sample was cooled and transferred to a sample vial for GC-MS/MS analysis.

#### MBA derivatization

In a vial, 100 µL of ethyl acetate and 100 µL of MBA were mixed. The vial was sealed and placed in an oven at 50 °C for 30 min. The sample was cooled and transferred to a sample vial for GC-MS/MS analysis.

### GC-MS/MS Conditions

#### Chromatographic conditions

A trace gas-chromatograph, TSQ Quantum, equipped with a TR-5MS column (30 m x 0.25 mm x 0.25 µm) was used for analysis. Helium (99.999%), with a constant flow rate of 1 mL/min, was used as the mobile phase. The inlet temperature was set to 260 °C, and 1 µL samples were injected splitlessly. The temperature gradient was set as follows: 70 °C for 1 min; temperature increase at a rate of 30 °C/min to 200 °C; increase at 3 °C/min to 245 °C; increase at 30 °C/min to 280 °C; hold at 280 °C for 3 min. The temperature for transmission was set at 280 °C, and the solvent delay was 5 min.

#### MS/MS conditions

**Figure 1:**
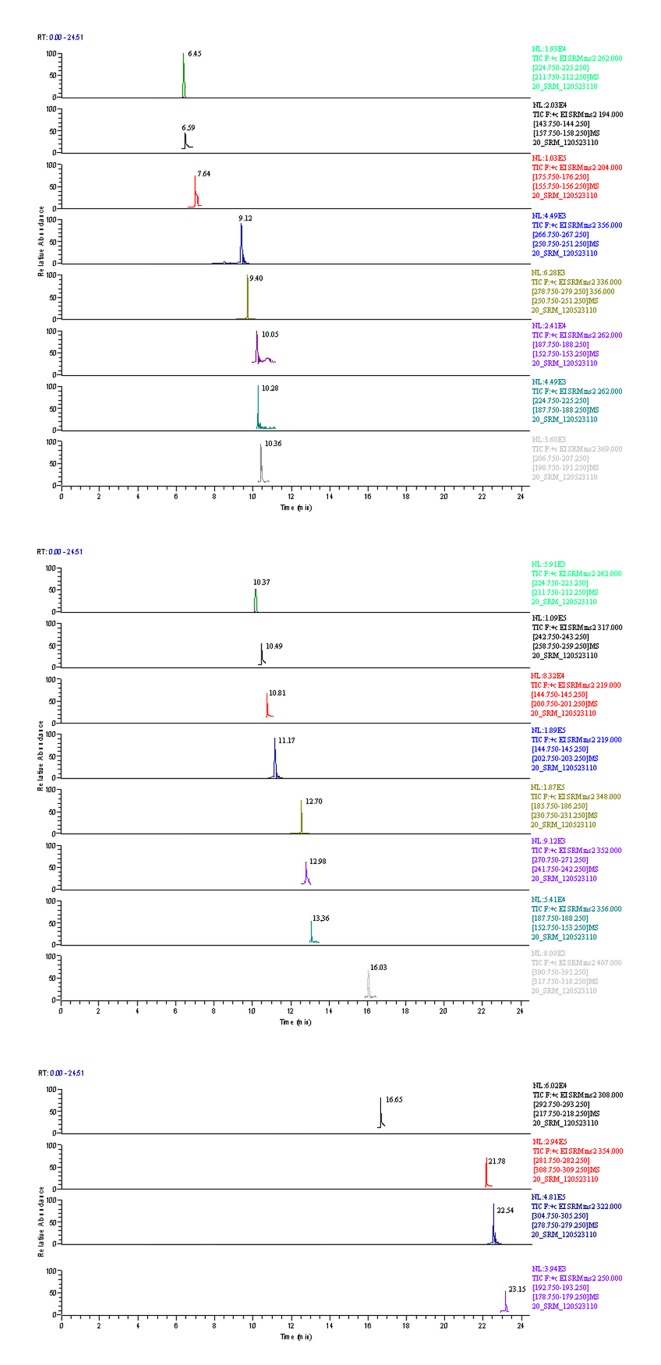
GC-MS/MS (SRM mode) chromatographs of 20 β-agonists.

A closed electron impact (CEI) ion source, with a source temperature of 230 °C, was used for ionization. Argon was used as the collision gas with a cell pressure of 1.5 mTorr. The FWHM peak width for Q1/Q3 was set at 0.7. The stimulating current was set at 50 µA. Segment and scan event modes were used, experimental parameters are listed in Table S1. Multiple reactor monitoring mode was used for the 20 analytes (parameters are shown in Table 1). The SRM chromatographs of the 20 β-agonists are shown in Figure 1.

**Table 1:** GC-MS/MS parameters for 20 β-agonists.

**β-agonist**	**BSTFA+TMCS**			**MSTFA**		
	**Retention Time (min)**	**Ion pairs used for identification (m/z)**	**Collision Energy (eV)**	**Retention Time (min)**	**Ion pairs used for identification (m/z)**	**Collision Energy (eV)**
Clorprenaline	6.45	262>225*	20	6.43	270>116*	15
		262>212			213>167	
Tulobuterol	6.59	194>144*	15	6.57	194>144*	15
		194>158			228>210	
Mabuterol	7.64	204>176*	15	7.62	277>200*	12
		204>156			296>204	
Metaproterenol	9.12	356>267*	15	10.57	355>281*	15
		356>251			355>239	
Terbutaline	9.40	336>279	20	9.35	356>267*	16
		356>251*			336>279	
Clenproperol	10.05	262>188*	15	11.80	262>188*	15
		262>153			212>182	
Cycloclenbuterol	10.28	262>225*	15	10.34	262>225	15
		262>188			243>187*	
Salbutamol	10.36	369>207*	15	10.30	369>207*	14
		369>191			369>191	
Clenbuterol	10.37	262>225*	20	10.34	246>220*	15
		262>212			262>228	
Salmeterol	10.49	317>243*	15	10.45	317>243*	15
		317>259			259>141	
Cimaterol	10.81	219>145*	15	10.77	219>203*	15
		219>201			221>166	
Cimbuterol	11.17	219>145	10	11.11	219>178*	15
		219>203*			234>160	
Penbutolol	12.70	348>186	15	12.72	348>249*	16
		348>231*			247>206	
Brombuterol	12.98	352>271*	20	12.91	352>272*	15
		352>242			264>185	
Clenisopenterol	13.36	262>188*	15	14.02	262>188*	15
		262>153			240>161	
Procaterol	16.03	407>391*	15	15.96	407>390*	15
		407>318			318>302	
Zilpaterol	16.65	308>293*	10	16.53	308>219*	18
		308>218			291>275	
Bambuterol	21.78	354>282*	15	21.71	354>309*	15
		354>309			439>354	
Fenoterol	22.54	322>305*	15	22.61	322>305*	15
		322>279			412>322	
Ractopamine	23.15	250>193*	15	23.13	267>193*	14
		250>179			250>206	

* The ion pair was used for quantification

## Results and Discussion

### Optimization of the mass spectrometer

The key to optimization is to select the appropriate ion pair for identification and quantification; ions with high abundance and signal intensities are preferred. As the type and signal intensities of fragment ions varies as a function of collision energy, it is essential to optimize this parameter to maximize the formation of high mass-to-charge ratio (m/z) ion pairs. In addition, according to the European Union Commission Decision 2002/657/EC [17], one precursor ion and two product ions were used to identify each compound, while only one ion pair was used for quantification. The optimized collision energies and respective ion pairs for the 20 derivatized β-agonists are given in Table 1. MBA derivatization yielded ion pairs with low signal intensities, and was not pursued further.

### Extraction solvent optimization

The highly polar β-agonists can be categorized as benzyl-ethanol amides, and are easily solubilized in acidic solution via formation of hydrogen bonds with the amine and hydroxyl functional groups. Various solvents were tested, but recoveries were highest with pH 4.8 sodium acetate buffer, as reported in Bulletin 1063-7-2008 [3]. Upon the addition of buffer, the analyte molecules were transferred into the aqueous layer, while nonpolar impurities such as fats were retained in the methanolic layer.

The mechanical shaking rate and shaking time also needed to be optimized. Vigorous shaking and long shaking durations enhanced extraction efficiencies but led to the generation of impurities; thus, a balance between extraction efficiency and impurity generation was determined. The highest recoveries were obtained when the liquid surface in motion was 1–2 times that of the still liquid surface, and the optimal shaking duration was 20 min.

### SPE column selection for purification

Three types of SPE columns were evaluated: a molecular imprinted polymer extraction column (MIP; β-agonists, 25 mg/10 mL, Lund, Sweden), an HLB column, and an MCX column. The MIP column exhibited high selectivity for several β-agonists, including clenbuterol, ractopamine, chlorprenaline and tulobuterol (Table 2), but also some of the lowest recoveries, including only 50.2% for procaterol [18]. The HLB column (60 mg/3 mL, waters OASIS) exhibited the largest range of recoveries, from 48.1 to 120.8% (Table 2).

**Table 2:** Recoveries (%) obtained from MCX, MIP, and HLB SPE columns for the 20 β-agonists analytes at concentration of 0.5 mg/kg.

**β-agonist**	**MCX**	**MIP**	**HLB**
Clorprenaline	90.4	99.8	88.3
Tulobuterol	90.7	92.4	88.1
Mabuterol	80.4	76.0	62.0
Metaproterenol	79.2	60.5	120.8
Terbutaline	80.5	55.1	74.7
Clenproperol	88.9	84.3	72.9
Cycloclenbuterol	88.1	70.9	65.8
Salbutamol	90.8	77.7	68.3
Clenbuterol	101.5	90.8	87.9
Salmeterol	80.0	53.2	60.4
Cimaterol	79.4	63.9	59.3
Cimbuterol	83.0	78.9	60.9
Penbutolol	89.6	77.2	68.3
Brombuterol	87.4	69.4	61.4
Clenisopenterol	100.9	78.3	79.3
Procaterol	93.5	50.2	80.8
Zilpaterol	79.0	67.5	48.9
Bambuterol	84.3	68.9	80.4
Fenoterol	81.6	59.1	49.0
Ractopamine	78.3	85.7	48.1

However the MCX (60 mg/3 mL, waters OASIS) column, in particular, showed relatively high recoveries for all 20 β-agonists, which ranged from 78.3 to 101.5% (Table 2). Addition of pH 4.8 sodium acetate buffer to feed acidifies the samples, resulting in protonated β-agonists. The Oasis MCX SPE column contains a combination reversed phase, strong cation-exchange polymeric sorbent which allows binding of basic analytes under acidic conditions. Moreover, the polar, amide-free MCX hydroxylated polymer column does not bind macromolecules, unlike typical matrices, and results in a reduction of ion suppression [8]. Because of the high recoveries attained, the MCX SPE column was used for further studies; the MCX column also notably outperforms the SCX (500 mg/3mL, Supelco) SPE column [12,13].

### Derivatization reaction parameterization

#### Experimental details and repeatability

The polar β-agonists do not volatilize easily, and require derivatization prior to GC-MS/MS analysis. For the derivatization process, it is essential to exclude water, because it can poison the derivatization reagents. To avoid water contamination, dry glassware, sample caps, and sealing film were used. Moreover, pre-drying glassware at 70 °C under nitrogen flow was found to improve the derivatization reactions.

**Figure 2:**
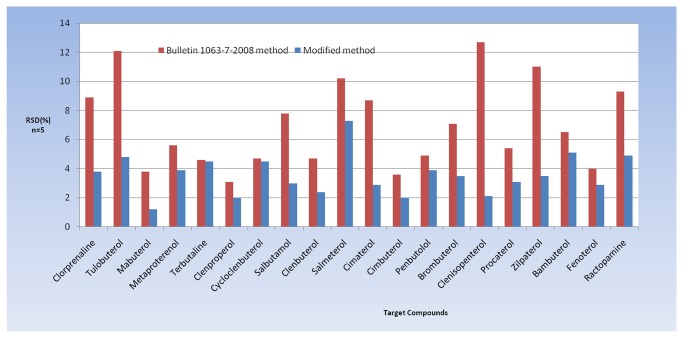
Repeatability of the reported method and the method described in Bulletin 1063-7-2008.

The repeatability of the reported method and the method described in Bulletin 1063-7-2008 were compared. Five replicates of blank feed samples were spiked with the 20 β-agonists (1 mg/kg). After MCX cleanup and BSTFA+TMCS derivatization, repeatability was calculated and expressed as the relative standard deviation (RSD). The method reported herein was found to have better repeatability (lower RSD) for all 20 β-agonists (Figure 2).

#### Products from the derivatization reactions

**Figure 3:**
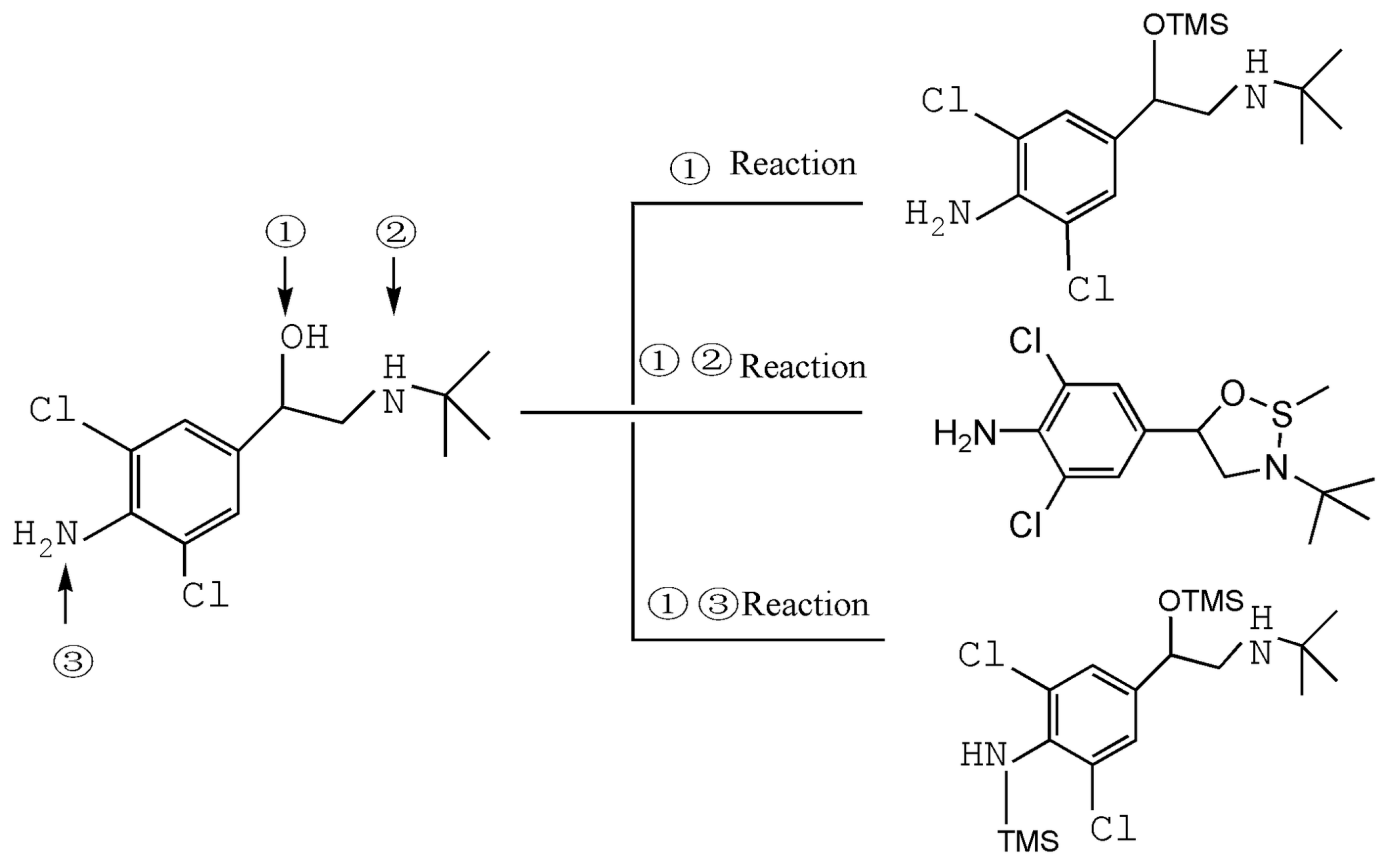
Three derivatization reactions of clenbuterol.

Three derivatization reagents, BSTFA+TMCS, MSTFA, and MBA, were evaluated. Reactions with BSTFA+TMCS and MSTFA resulted in the formation of high intensity derivatives. Derivatization of clenbuterol, for example, gave a major product of O-TMS and a side product of N with a ratio of ~3:1, which is consistent with previous reports [19–21]. The data indicate that O-TMS derivatization at the hydroxyl position of β-ethanolamine (at position 1 as shown in Figure 3) represents the major product, and derivization at the aromatic amino group yields the minor N,O-TMS product (at positions 1 and 3 as shown in Figure 3). The major product from MBA derivatization is simply methyl borate (at position 1 and 2 as shown in Figure 3).

#### The influence of derivatization time and temperature

**Figure 4:**
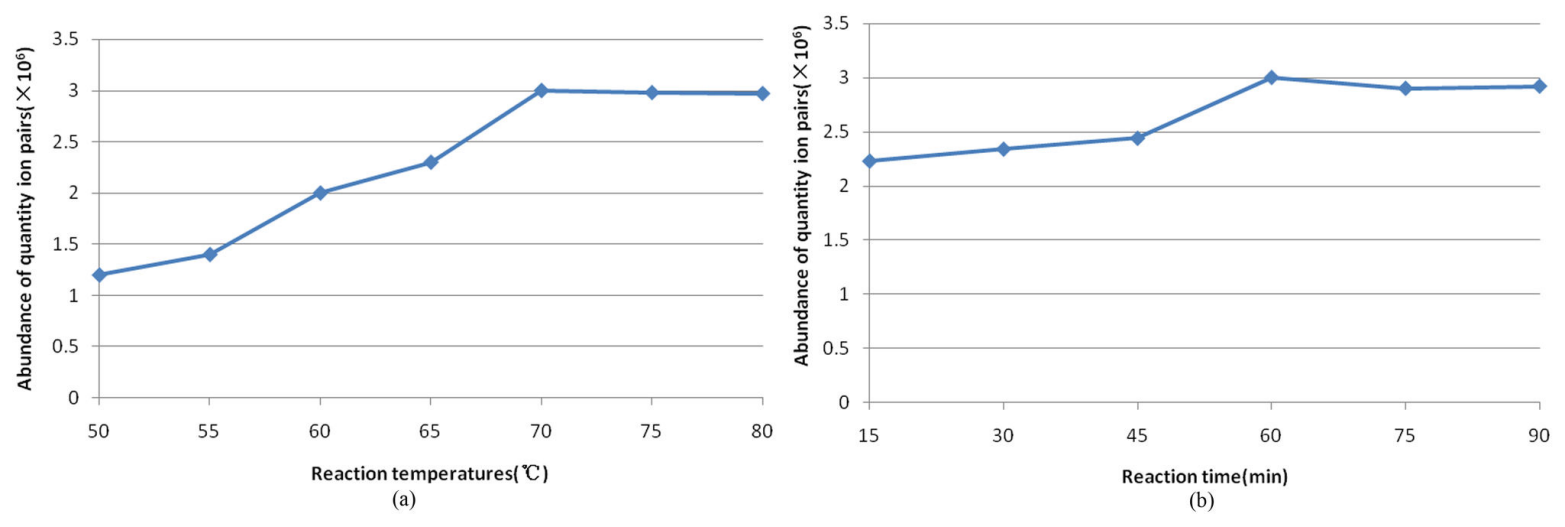
Dependence of reaction (a) temperature and (b) time on quantitative ion intensity.

A variety of reaction temperatures (50, 60, 70 and 80 °C) and times (15,30,45,60,75, and 90 min) were evaluated to determine the optimal reaction conditions. Conversion rates for the three derivatization reactions increased with increased reaction temperature and time initially, and then stabilized (Figure 4). For this study, a reaction temperature of 70 °C for 60 min was ideal.

#### Stability studies

**Figure 5:**
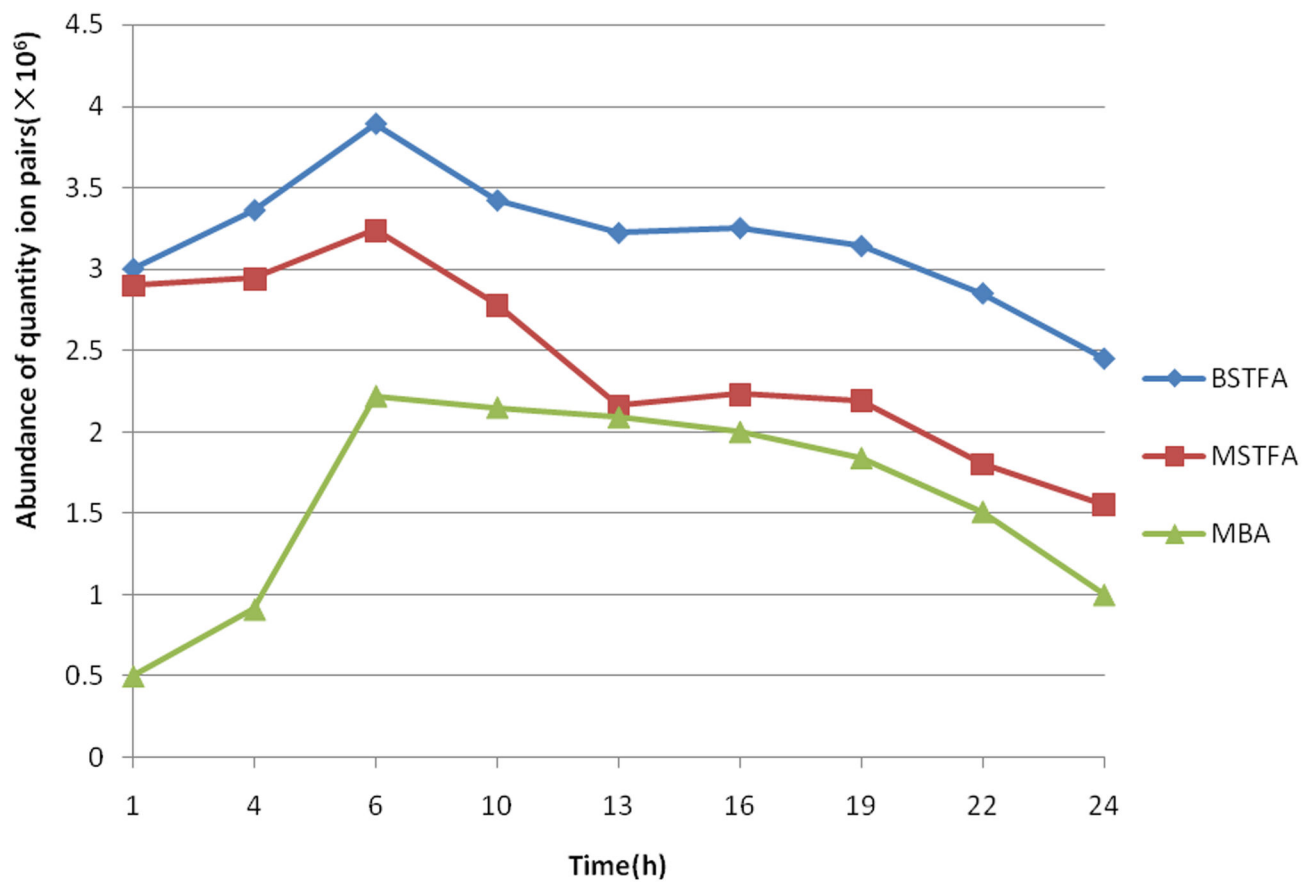
Quantitative ion intensities obtained for the three derivatization products.

Following the derivatization reactions, the samples were cooled to room temperature, and product stability was tested at various time intervals (1, 4,6,10,13,16,19,22 and 24 h) via GC-MS/MS analysis. A similar trend in product stability was observed for all 20 β-agonists for the three types of derivatization (Figure 5). Initially, the intensity of the quantitative ion peaks increased, and then they decreased (Figure 5). The maximum intensity for the three derivatization methods was found at 6 h, and the increase in intensity over this period may be attributable to an increase in concentration caused by solvent evaporation. After 6 h, the peaks decrease which is likely due to decomposition of the products. The BSTFA derivatization products decomposed faster than those formed in the MSTFA and MBA reactions (Figure 5), suggesting that the BSTFA products are relatively unstable.

The intensity of the quantitative ion pairs followed this order: BSTFA+TMCS ^≈^ MSTFA > MBA. Since BSTFA+TMCS and MSTFA outperformed MBA in terms of ion signal intensity, these derivatization methods were used for subsequent experiments. Method validation, to elaborate the accuracy and reproducibility of the derivatization reactions, using GC-MS/MS was then performed.

### Method Validation

#### Matrix effects, limit of detection (LOD), limit of quantification (LOQ) and linear range

Matrix effects need to be considered because feed is a complex, interference-laden medium. In this study, it was found that 5 different feed matrixes (formulated, complete, concentrated, mixed, and compound-premix feed) have similar matrix suppression effects for the 20 β-agonists. To avoid matrix effects, the spiked samples were compared with blank feed samples.

The LOD was determined using blank feed samples spiked with decreasing concentrations (50, 25, 10, 5, and 2.5 ng/ml) of the β-agonists. The samples were subjected to the extraction, purification, and derivatization procedure and analyzed via GC-MS/MS. The LOD was determined to be 0.01 mg/kg, which was estimated by establishing the minimum concentration at which the analytes could be detected with a signal to noise ratio greater than three to one (S/N ≥ 3). The limit of quantitation (LOQ) was found to be 0.05 mg/kg (S/N ≥ 10).

The 5 blank feed matrixes were spiked with β-agonists with concentrations in the range of 0.05 to 1 mg/kg and calibration curves and the regression equation were constructed (Table 3). Correlation coefficients (R^2^) for the calibration curves were greater than 0.99, and during analysis, if the response exceeded the linear range, the sample was diluted appropriately and re-tested.

**Table 3:** Regression equation and correlation efficiency for 20 β-receptor agonists.

**β-receptor agonists**	**Regression equation** ^*^	**Correlation coefficient**
Clorprenaline	Y=12984.5X+45.7	0.9931
Tulobuterol	Y=329.5X+98.1	0.9941
Mabuterol	Y=3904. X+12.9	0.9924
Metaproterenol	Y=934.2X+75.8	0.9906
Terbutaline	Y=18994.5X+63.0	0.9928
Clenproperol	Y=28954.5X+95.4	0.9973
Cycloclenbuterol	Y=7980.6X+17.1	0.9943
Salbutamol	Y=43793.5X+20.4	0.9957
Clenbuterol	Y=40821.9X+37.9	0.9978
Salmeterol	Y=37583.5X+90.9	0.9931
Cimaterol	Y=9549.0X+39.3	0.9961
Cimbuterol	Y=22463.2X+18.9	0.9989
Penbutolol	Y=10849.5X+75.7	0.9953
Brombuterol	Y=28548.5X+20.3	0.9988
Clenisopenterol	Y=189.0X+105.5	0.9973
Procaterol	Y=3543.4X+45.8	0.9921
Zilpaterol	Y=493.6X+43.2	0.9917
Bambuterol	Y=15964.4X+29.6	0.9929
Fenoterol	Y=5639.2X+85.4	0.9918
Ractopamine	Y=25343.5X+49.l	0.9901

* The 5 blank feed matrixes were spiked with β-agonists with concentrations in the range of 0.05 to 1 mg/kg

In summary, the new quantification method showed similar linearity, LOD, and LOQ as state-of-the-art methods, and is as sensitive as the LC-MS/MS method reported in Bulletin 1063-6-2008 by the Chinese department of agriculture (LOD 0.01mg/kg LOQ 0.05mg/kg) and some methods established worldwide [7,10–12,22,23].

#### Repeatability, recoveries, and precision

Repeatability was evaluated for 5 types of feed samples spiked at 3 concentrations (0.05, 0.1, and 1 mg/kg) after analysis and again on the same day (Table 4); the relative standard deviation (RSD) for the quantitative ion peak areas of three replicate measurements was used for assessment. Moreover, the intermediate precision was determined by analysis of the spiked samples (0.05, 0.1, 1 mg/kg) over three consecutive days (Table 4); precision was also expressed as the relative standard deviation (RSD).

**Table 4:** Intraday mean recoveries (%; n = 3), repeatability (%; n =3), and intermediate precision (%; n = 9) of 20 β-agonists at three spike concentrations in formulated feed determined by GC-MS/MS.

**β-agonist**	**Spike concentration**	**Intraday Mean**			**Intermediate**
	**(µg/g)**	**Recovery (Repeatability)**			**precision**
		Day 1	Day 2	Day 3	
Clorprenaline	0.05	79.5 (3.0)	80.3 (4.8)	81.2 (5.1)	13.4
	0.1	78.0 (4.5)	80.2 (4.7)	79.6 (4.1)	10.3
	1	85.3 (3.8)	84.1 (9.1)	88.1 (1.2)	12.7
Tulobuterol	0.05	101.8 (6.7)	96.5 (7.2)	95.4 (6.9)	13.0
	0.1	91.5 (2.5)	92.3 (5.9)	90.8 (8.3)	10.9
	1	94.4 (4.8)	90.1 (9.2)	95.5 (4.0)	8.6
Mabuterol	0.05	77.5 (2.9)	79.0 (1.2)	79.2 (7.3)	12.4
	0.1	85.4 (2.8)	79.2 (5.4)	80.9 (7.3)	7.3
	1	79.6 (1.2)	80.1 (3.9)	81.2 (2.1)	9.6
Metaproterenol	0.05	80.5 (2.1)	80.5 (1.8)	78.2 (2.4)	11.9
	0.1	83.7 (4.3)	88.1 (4.4)	85.0 (3.9)	10.0
	1	82.6 (3.9)	81.6 (8.2)	83.3 (1.9)	13.6
Terbutaline	0.05	85.4 (3.9)	82.1 (4.1)	82.0 (3.2)	4.9
	0.1	88.9 (2.8)	84.5 (3.9)	85.1 (4.2)	7.3
	1	87.6 (4.5)	88.2 (2.3)	86.0 (7.4)	10.3
Clenproperol	0.05	80.1 (3.1)	78.3 (2.8)	77.3 (2.9)	5.8
	0.1	82.4 (4.8)	88.6 (1.2)	83.1 (8.6)	13.0
	1	85.0 (2.0)	87.7 (7.2)	82.2 (3.5)	8.8
Cycloclenbuterol	0.05	78.4 (2.6)	80.4 (8.6)	77.2 (1.2)	13.8
	0.1	78.0 (3.7)	83.4 (9.1)	77.9 (1.9)	7.5
	1	84.1 (4.5)	79.6 (3.3)	87.0 (3.9)	8.1
Salbutamol	0.05	101.5 (1.3)	97.1 (8.9)	102.1 (6.8)	8.8
	0.1	98.8 (4.1)	91.2 (4.4)	90.4 (5.0)	13.5
	1	99.3 (3.0)	91.2 (7.9)	88.4 (8.4)	10.5
Clenbuterol	0.05	95.7 (3.1)	95.5 (8.6)	98.1 (2.0)	13.3
	0.1	102.4 (5.9)	100.1 (4.3)	99.1 (4.9)	7.5
	1	99.7 (2.4)	100.1 (1.6)	95.4 (5.0)	12.7
Salmeterol	0.05	89.5 (6.3)	94.3 (4.0)	89.1 (2.3)	10.1
	0.1	85.5 (4.4)	79.2 (2.3)	77.1 (3.2)	6.8
	1	90.8 (7.3)	82.5 (2.1)	88.5 (5.5)	8.1
Cimaterol	0.05	78.4 (3.1)	77.1 (1.2)	78.0 (3.1)	12.9
	0.1	78.5 (6.5)	79.1 (5.8)	81.3 (6.1)	12.0
	1	85.0 (2.9)	81.1 (7.8)	84.3 (4.7)	9.8
Cimbuterol	0.05	80.1 (5.3)	88.3 (1.2)	85.5 (3.4)	10.2
	0.1	83.7 (3.3)	82.1 (2.6)	85.1 (2.6)	6.9
	1	83.2 (2.0)	85.4 (9.1)	88.3 (4.2)	12.1
Penbutolol	0.05	88.7 (6.1)	79.1 (5.1)	80.8 (5.1)	9.2
	0.1	89.4 (4.4)	83.4 (3.9)	88.3 (4.0)	12.9
	1	90.3 (3.9)	81.3 (2.0)	88.6 (2.9)	7.4
Brombuterol	0.05	90.8 (2.9)	95.5 (4.1)	88.7 (3.0)	11.8
	0.1	88.5 (4.5)	82.5 (3.4)	85.1 (4.1)	12.9
	1	91.4 (3.5)	89.3 (4.1)	91.4 (8.9)	10.9
Clenisopenterol	0.05	78.8 (3.9)	81.0 (2.1)	83.1 (2.0)	13.0
	0.1	85.6 (2.1)	83.1 (6.9)	79.0 (1.1)	8.1
	1	78.2 (2.1)	80.1 (1.9)	79.0 (6.9)	9.4
Procaterol	0.05	85.5 (1.9)	79.1 (2.0)	81.8 (1.8)	7.2
	0.1	87.6 (1.9)	88.1 (5.1)	85.2 (6.4)	11.9
	1	91.7 (3.1)	88.9 (2.8)	87.6 (2.5)	4.9
Zilpaterol	0.05	90.4 (4.9)	82.5 (4.1)	88.4 (3.4)	8.1
	0.1	88.6 (8.9)	80.1 (5.4)	85.3 (2.2)	12.5
	1	94.2 (3.5)	95.5 (7.9)	95.4 (3.1)	13.8
Bambuterol	0.05	85.6 (4.8)	82.2 (4.6)	83.3 (8.9)	12.4
	0.1	87.6 (1.4)	81.2 (3.2)	83.2 (3.0)	13.1
	1	92.3 (5.1)	91.0 (4.9)	89.4 (7.8)	10.5
Fenoterol	0.05	80.5 (5.5)	79.1 (1.5)	88.3 (5.0)	5.3
	0.1	78.9 (3.0)	80.0 (3.2)	81.1 (3.4)	13.1
	1	85.6 (2.9)	79.4 (7.5)	80.3 (3.2)	10.9
Ractopamine	0.05	75.6 (8.8)	80.3 (9.0)	77.8 (7.3)	8.7
	0.1	77.3 (3.4)	81.1 (9.3)	79.2 (9.3)	9.4
	1	80.5 (4.9)	82.2 (6.7)	79.2 (2.5)	9.6

Mean recoveries ranged from 75.6 to 102.4%, repeatability ranged from 1.2 to 9.4%, and intermediate precisions were lower than 13.8% for all of the samples tested. The results obtained from the formulated feed samples are summarized in Table 4. The recovery and repeatability values obtained were comparable with those from the standard method described in Bulletin 1063-6-2008 by the Chinese department of agriculture (80 ± 20% recoveries; 0.05–1.0 mg/kg; repeatability ≤ 20%) and other international studies [7,10–12,22,23].

#### Stability

Stability was determined via two methods with three replicates. In the first method, the methanolic standard solutions, stored in the dark at -20 °C, were analyzed weekly by UPLC and the obtained peak areas were compared with the peak area obtained from the freshly prepared solutions (t = 0 min); a peak area between 95 and 105% of the initial area was defined as acceptable [24]. For the second method, 6 feed matrixes, fortified with the 20 β-agonists (0.05 mg/kg) stored at -20 °C, were analyzed after 3, 7, 14, and 30 days; recoveries of the 20 β-agonists over this time period did not change.

### Application of the method

Under the optimized conditions, the method reported herein was applied to analyze 50 actual feed samples commercially available in China (number FXPC01-50). Quality control was achieved by spiking (0.4 mg/kg) blank feed samples. Recovery rates for the quality control experiments ranged from 80.3 to 98.7%. The concentration of clenbuterol in sample FXPC03 was 0.18 mg/kg, while the concentration of cycloclenbuterol in FXPC08 sample was 0.14 mg/kg.

## Conclusions

A method to quantify 20 β-agonists in feed via chemical derivatization followed by gas chromatography tandem mass spectrometry analysis was developed. The method, used to identify 20 β-agonists simultaneously in actual feed samples, is precise and accurate, and represents an excellent complement to existing quantification methods. The method reported herein was validated in terms of sensitivity, accuracy, intraday and interday precision, as well as linearity in accordance with European Commission Decision 2002/657/EC [17]. Additionally, the method has been checked and applied in “the check test of the forbidden drug in Chinese Feed Quality Inspection System” (2012) which was under the auspices of the Chinese Department of Agriculture.”

## Supporting Information

Table S1 **Parameters of scan segment and scan events.**
(DOC)
