# Supplementary material for: Simultaneous Identification and Quantification of 20 β-Receptor Agonists in Feed Using Gas Chromatography-Tandem Mass Spectrometry
Source: PLoS One. 2013 Oct 3;8(10):e76400. doi: 10.1371/journal.pone.0076400 (PMC3789743; doi:10.1371/journal.pone.0076400)
Supplement: Table S1 — Parameters of scan segment and scan events. (DOC) [file pone.0076400.s001.doc]

**Table S1.** Parameters of scan segment and scan events

| **Scan Segment** | **1** | **2** | **3** | **4** | **5** | **6** | **7** | **8** | **9** |
| --- | --- | --- | --- | --- | --- | --- | --- | --- | --- |
| **Duration（min）** | 5.00 | 2.00 | 1.00 | 1.70 | 1.30 | 1.00 | 2.00 | 3.50 | 6.50 |
| **Scan Event** | 0 | 2 | 1 | 2 | 6 | 1 | 2 | 1 | 2 |
